# Association between *JAK2*^*V617F*^ variable allele frequency and risk of thrombotic events in patients with myeloproliferative neoplasms

**DOI:** 10.1007/s11845-024-03776-5

**Published:** 2024-08-14

**Authors:** Ryan Brown, Joanna Jasiakiewicz, Victoria Greer, Andrew Hindley, Katie McDowell, Eadaoin Devlin, Kathryn Clarke, Frances Buckley, Clare Crean, Julie McGimpsey, Robert J. G. Cuthbert, Nick Cunningham, Claire Arnold, Damian Finnegan, Gary Benson, Mary Frances McMullin, Mark A. Catherwood

**Affiliations:** 1 Haematology, Belfast City Hospital, BHSCT, Belfast, Northern Ireland UK; 2 Haematology Department, NHS Highland, Inverness, Scotland UK; 3 Centre for Medical Education (CME), Queen’s University Belfast, Belfast, Northern Ireland UK

**Keywords:** *JAK2*, Myeloproliferative neoplasm, Thrombosis, Variable allele frequency

## Abstract

**Background:**

Myeloproliferative neoplasms (MPNs) are a group of chronic disorders of the bone marrow characterised by the overproduction of clonal myeloid stem cells. The most common driver mutation found in MPNs is a point mutation on exon 14 of the *JAK2* gene, *JAK2*^*V617F*^*.* Various studies have suggested that measuring the variable allele frequency (VAF) of *JAK2*^*V617F*^ may provide useful insight regarding diagnosis, treatment, risks and outcomes in MPN patients. In particular, *JAK2*^*V617F*^ has been associated with increased risk of thrombotic events, a leading cause of mortality in MPNs.

**Aims:**

The aim of this study was to determine if *JAK2*^*V617F*^ VAF was associated with clinical outcomes in patients with MPN.

**Methods:**

*JAK2*^*V617F*^ VAF was determined by quantitative PCR (qPCR) in a cohort of 159 newly diagnosed MPN patients, and the association of *JAK2*^*V617F*^ VAF and risk of thrombosis was examined in this cohort.

**Results:**

We observed a significantly higher *JAK2*^*V617F*^ VAF in PV and PMF versus ET. A significant association was observed between *JAK2*^*V617F*^ VAF and risk of thrombotic events. When patients were stratified by thrombotic events prior to and post diagnosis, an association with *JAK2*^*V617F*^ VAF was only observed with post diagnosis thrombotic events. Of note, these associations were not observed when looking at each MPN subtype in isolation.

**Conclusions:**

We have shown that a higher *JAK2*^*V617F*^ VAF is associated with thrombotic events post MPN diagnosis. *JAK2*^*V617F*^ VAF may therefore provide a valuable prognostic indicator for risk of thrombosis in MPNs.

## Introduction

Myeloproliferative neoplasms (MPNs) are a group of chronic disorders of the bone marrow characterised by overproduction of clonal myeloid stem cells [1]. MPNs are classified into three subtypes based on clinical and molecular features—polycythaemia vera (PV), essential thrombocythaemia (ET) and primary myelofibrosis (PMF) [1–3]. The most common genetic mutation in all MPNs is *JAK2*^*V617F*^. It is present in up to 95% of PV cases and 50% of ET and PMF cases and has importance in terms of diagnosis, treatment, outcomes and risks [4].

In addition to assessing the *JAK2*^*V617F*^ mutational status in MPNs patients, there is an increasing interest in measuring the *JAK2*^*V617F*^ variable allele frequency (VAF) as it has been proposed that the mutation burden may have significant clinical implications [5]. Indeed, previous studies in patients with PV demonstrated increased risk of myelofibrotic transformation in participants with a higher *JAK2*^*V617F*^VAF [6, 7]. Furthermore, it has been shown that PV patients with a *JAK2*^*V617F*^ allele burden of 58% or more had significantly reduced overall survival [8]. Similarly, a retrospective study in patients with ET observed an increased risk of thrombotic events in patients with a mutational burden higher than 30% [8]. Another study in MPN patients with *JAK2*^*V617F*^showed an association between VAF and increased risk of venous thromboembolism but no association with overall cardiovascular events [9].

These studies highlight the potential use of *JAK2*^*V617F*^ VAF in predicting risks and outcomes in patients with MPNs. Therefore, our aim was to investigate the role of *JAK2*^*V617F*^ VAF in a cohort of newly diagnosed MPN patients and to examine the association of *JAK2*^*V617F*^ VAF and risk of thrombosis in this cohort.

## Methods

A real-world cohort of 63 patients with a diagnosis of PV, 89 patients with diagnosis of ET and 7 patients with diagnosis of PMF were included, between the years of 2019 and 2021 according to the WHO classification criteria. Peripheral blood samples from each patient were tested for the *JAK2*^*V617F*^ mutation, and a retrospective audit of diagnosis and outcomes was undertaken. All samples were acquired pre-diagnosis. The management of anticoagulants and cytoreduction treatments following diagnosis was appropriately tailored based on the patient’s risk group.

### Data collection

Demographic characteristics (age and gender) and clinical information (haemoglobin, haematocrit, platelets, total white cell count (WCC), the presence of splenomegaly, thrombosis before diagnosis, bleeding before diagnosis, thrombosis after diagnosis, bleeding after diagnosis) were determined.

### Quantitative PCR

Quantitative PCR (qPCR) was performed on DNA extracted from peripheral blood using a Roche LightCycler 480 PCR system with custom PCR primers for *JAK2* WT reverse (Eurofins), *JAK2*^*V617F*^ reverse (Eurogentec), common JAK2 forward (Eurofins) and custom *JAK2*^*V617F*^ probe (Thermo Fisher). WT and *JAK2*^*V617F*^ copy number were determined by comparison to a standard curve. Standard curves for WT and *JAK2*^*V617F*^ were generated from the WHO 1st International Reference Panel for Genomic *JAK2*^*V617F*^ 16/120. VAF was determined by calculating the percentage of *JAK2*^V617F^ reads to total *JAK2* (^V617F^ and ^WT^) reads. The limit of detection for this assay was 0.03% VAF.

### Statistics

All data were analysed using Excel and are reported as mean ± SD. Statistical analyses were performed using Student’s *t* test, and *P* < 0.05 was accepted to indicate statistical significance.

## Results

Clinical characteristics and haematological values were assessed in 63 PV, 89 ET and 7 PMF patients (Table 1). The average age at diagnosis was 71 (range 37–94) for PV patients, 69 (range 29–91) for ET patients and 78 (range 66–93) for PMF patients. Within the PV patient group 55.6% were male, 33.7% of ET patients were male, and 71.4% of PMF patients were male. Mean haemoglobin concentrations were significantly increased in PV 180 g/l (range 139–228), versus ET 139.9 g/l (range 93–169) (*P* < 0.001) and PMF 99.5 g/l (range 68–131), (*P* < 0.001). Similarly, the mean haematocrit value was significantly increased in PV 54.6% (range 46–67) versus ET 42.6% (range 29–50). However, no significant differences were observed between PMF 20.8% (range 20.8–20.8) and ET or PV. Platelets were significantly elevated in ET patients versus PV and ET patients with mean platelet counts of 719.6 × 10^9^/L (range 68–1513) in ET, 502.0 × 10^9^/L (range 129–1112) in PV and 333.2 × 10^9^/L (range 83–528) in PMF. Total WCC was significantly raised in PV 13.3 × 10^9^/L versus ET 9.9 × 10^9^/L and PMF 12.5 × 10^9^/L. Splenomegaly was present in 57.1% of patients with PMF, 12.9% of patients with PV and 3.3% of patients with ET. Prior thrombotic events were observed in a greater proportion of PV cases (42.9%) when compared with ET cases (23.6%). As expected, a lower percentage of patients experienced thrombotic events post diagnosis than prior to diagnosis with 11.1% of PV and 2.2% of ET cases experiencing thrombotic events post diagnosis. No thrombotic events prior or post diagnosis were observed in PMF patients included in this study. No cases of major bleeding prior or post diagnosis were recorded in this subset of PV, ET or PMF patients.

**Table 1:** Demographic data for cases included in the study. *n* = 63 (PV), 89 (ET) and 7 (PMF)

	PV	ET	PMF
Age, years (range)	71 (37 – 94)	69 (29 – 91)	78 (66 – 93)
Male gender, (N) %	35 (55.6)	30 (33.7)	5 (71.4)
Haemoglobin g/dl (range)	180 (139—228)	140 (93 – 169)	99.5 (68 – 131)
Haematocrit, % (range)	54.6 (46 – 67)	42.6 (29 – 50.0)	20.8 (20.8 – 20.8)
Platelets × 10^9^/L (range)	502 (129—1112)	719.6 (68—1513)	333.2 (83—528)
White cell count × 10^9^/L	13.3 (5.7—29)	9.9 (3.0—21.0)	12.5 (6.0—16.0)
Presence of Splenomegaly, N (%)	8 (12.9)	3 (3.3)	4 (57.1)
Thrombosis before diagnosis, N (%)	27 (42.9)	21 (23.6)	0 (0)
Bleeding before diagnosis, N (%)	0 (0)	0 (0)	0 (0)
Thrombosis after diagnosis, N (%)	7 (11.1)	2 (2.2)	0(0)
Bleeding after diagnosis, N (%)	0 (0)	0 (0)	0 (0)

**Fig. 1:**
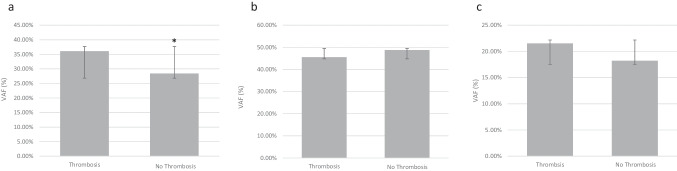
Comparison of *JAK2*^*V617F*^ variant allele frequency (VAF) between **a** all cases, **b** polycythaemia vera (PV) cases, and **c** essential thrombocythaemia (ET) cases with, and without thrombotic events. *n* = 22–103 per group. *P < 0.05

**Fig. 2:**
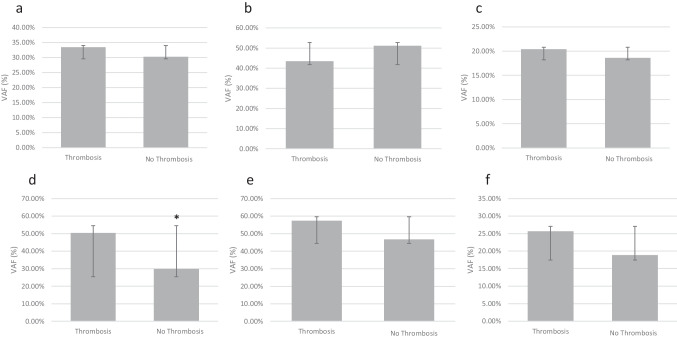
Comparison of *JAK2*^*V617F*^ VAF between **a**, **d** all cases, **b**, **e** polycythaemia vera (PV) cases and **c**, **f** essential thrombocythaemia (ET) cases with thrombotic events prior to (**a**–**c**) or post (**d**–**f**) diagnosis. *n* = 2–143 per group, *P < 0.05

*JAK2*^*V617F*^ VAF was assessed by QPCR in patients with PV, ET or PMF. Mean *JAK2*^*V617F*^ VAF was significantly higher in PV 47.83% (range 1.48–91.85) vs ET 19.02% (range 0.9–65.96), (*P* < 0.001) patients. A significant increase in *JAK2*^*V617F*^ VAF was also observed in ET vs PMF 41.2% (range 17.9–71.1) (*P* < 0.001). However, no statistically significant difference in *JAK2*^*V617F*^ VAF was observed between the PMF and PV groups (Table 2). Patients who had experienced thrombotic events had significantly higher *JAK2*^*V617F*^ VAF than those that had not experienced any thrombotic events (Fig. 1a). This significant difference was not observed when results were broken down into MPN subtypes (Fig. 1b–c). We next compared *JAK2*^*V617F*^ VAF in patients who had experienced thrombotic events either prior to or post diagnosis (Fig. 2a–f). *JAK2*^*V617F*^ VAF was not associated with a higher risk of prior thrombosis in either PV or ET patients independently or combined in our cohort (Fig. 2a–c). However, a significant increase in *JAK2*^*V617F*^ VAF was observed in patients that had experienced thrombotic events post diagnosis versus those that experienced no thrombotic events (Fig. 2d). This significant difference was not observed when results were broken down into MPN subtypes (Fig. 2e–f). We next compared clinical and haematological characteristics between MPN patients with and without thrombotic events prior to and post diagnosis (Tables 3 and 4). There were no significant differences in the age of diagnosis, haemoglobin concentration, haematocrit, platelet count or total white cell count between patients with or without thrombotic events prior to or post diagnosis.

**Table 2:** Variable allele frequency (VAF) of *JAK2*^*V617F*^ mutation in myeloproliferative neoplasms (MPN)

Diagnosis (n)	PV (63)	ET (89)	PMF (7)	Statistics
*JAK2*^*V617F*^ VAF (%) Mean (range)	47.8 (1.48—91.85)	19.0 (0.9—65.96)	41.20 (17.9—71.1)	ET vs PV *P* < 0.001
				ET vs PMF *P* < 0.001

**Table 3:** Number of thrombotic events in myeloproliferative neoplasms (MPN) prior to and post diagnosis

	PV	ET	PMF
No Thrombotic Events, N (%)	29 (46.03)	66 (74.15)	7 (100)
Prior Dx Thrombotic Event, N (%)	27 (42.86)	21 (23.60)	0 (0)
Post Dx Thrombotic Event, N (%)	7 (11.11)	2 (2.25)	0 (0)

**Table 4:** Demographic data comparing cases with and without thrombotic events prior to diagnosis

Prior	All Patients	Patients with prior thrombosis	Patients without prior thrombosis
Age, years (range)	70 (29—94)	72 (40—94)	69 (29—93)
Male gender, N (%)	68 (42.77)	28 (58.33)	40 (36.04)
Haemoglobin g/dl (range)	153.6 (68—228)	158.8 (111—214)	151.2 (68—228)
Haematocrit, % (range)	48.2 (20.8—62)	49 (34—62)	47.8 (20.8—67)
Platelets × 10^9^/L	621.6 (68—1513)	646 (68—1513)	610.2 (83—1436)
White cell count × 10^9^/L	11.28 (3—29)	12 (6—27)	10.9 (3—29)
Presence of Splenomegaly, N (%)	15 (9.43)	3 (6.25)	12 (10.81)
VAF, % (range)	31.4 (0.9—91.9)	32.5 (2.8—88.7)	30.9 (2.8—91.9)

## Discussion

We present here a cohort of MPN patients harbouring the V617F mutation in the *JAK2* gene. We have compared clinical characteristics and VAF between patients with PV, ET and PMF (Tables 1 and 2), examined the rate of thrombotic events in each MPN subtype (Table 3), examined the association of VAF with risk of experiencing thrombotic events (Figs. 1 and 2) and compared the clinical characteristics of patients that experienced or did not experience thrombotic events prior to or post diagnosis (Table 4 and 5).

**Table 5:** Demographic data comparing cases with and without thrombotic events post diagnosis

Post	All Patients	Patients with post thrombosis	Patients without post thrombosis
Age, years (range)	70 (29—94)	73 (61—84)	70 (29—94)
Male gender, N (%)	68 (42.77)	4 (44.44)	64 (42.67)
Haemoglobin g/dl (range)	153.6 (68—228)	168.8 (130—198)	152.8 (68—228)
Haematocrit, % (range)	48.2 (20.8—62)	54.3 (47—59)	47.9 (20.8—67)
Platelets × 10^9^/L	621.6 (68—1513)	525 (226 -1153)	627 (68—1513)
White cell count × 10^9^/L	11.28 (3—29)	12.3 (7.6—18.7)	11.2 (3—29)
Presence of Splenomegaly, N (%)	15 (9.43)	1 (11.11)	14 (9.33)
VAF, % (range)	31.4 (0.9—91.9)	50.3 (1.5—91.9)	30.3 (0.9—89.8)

We have shown that significant differences in the clinical characteristics are present between patients with PV, ET and PMF. Namely, the mean haemoglobin concentration and haematocrit are significantly higher in patients with PV, whilst platelet counts are significantly higher in patients with ET. These results are in line with current diagnostic criteria for the classification of MPNs. Diagnosis of PV requires an elevated haemoglobin concentration or elevated haematocrit as a major criterion, whilst diagnosis of ET requires an elevated platelet count [10]. Whilst the mean age at diagnosis is similar for all three of the MPN sub-groups, there was a significant skew in gender in our ET cohort with 76.3% being female. This is consistent with previous published studies indicating a predominance of females in the ET population [11, 12]. The percentage of patients with splenomegaly is significantly higher in the PMF group compared to both the ET and PV group. As expected, the mean *JAK2*^*V617F*^ VAF was found to be significantly higher in the PV and PMF group than in the ET group.

Within our cohort, we observed significantly lower *JAK2*^*V617F*^ VAF in ET versus PV and PMF (Table 2). This is consistent with previously published data showing a mean VAF below 20% for ET and above 40% for PV [13]. Interestingly, *in vivo* studies have demonstrated that low level *JAK2*^*V617F*^ expression in transgenic mice results in an ET-like phenotype with marked thrombocytosis, whilst high *JAK2*^*V617F*^ expression results in erythrocytosis as seen in PV [14]. This suggests that *JAK2*^*V617F*^ VAF may play a direct role in MPN pathogenesis. The prognostic impact of *JAK2*^*V617F*^ VAF in MPN has been documented in a number of previous studies showing increased risk of thrombotic events, higher rate of progression to secondary myelofibrosis and reduced overall survival in patients with higher *JAK2*^*V617F*^ VAF [6–9]. As such, *JAK2*^*V617F*^ genotype has been included in the International Prognostic Score of thrombosis in ET (IPSET-thrombosis) [15]. Indeed, vascular thrombotic complications are a major cause of morbidity and mortality in MPNs [16].

In our cohort, we observed a significantly higher *JAK2*^*V617F*^ VAF in patients that had a thrombotic event versus those that did not (Fig. 1). This was due to a significant increase in the *JAK2*^*V617F*^ VAF in patients that had thrombotic events post diagnosis. No associations were observed between thrombotic events prior to diagnosis and *JAK2*^*V617F*^ VAF in this study (Fig. 2). The majority of previous studies have not separated thrombotic events into prior to or post diagnosis; however, one study showed that a VAF above 75% is associated with increased risk of thrombosis post diagnosis [17]. The underlying mechanism by which *JAK2*^*V617F*^ VAF modulates post diagnosis thrombotic risk specifically is not clear. However, post diagnosis other factors known to contribute to thrombotic risk including raised haematocrit and total WCC are more closely controlled potentially increasing the proportional contribution of *JAK2*^*V617F*^ VAF. These data highlight the importance of addressing clonal expansion of *JAK2*^*V617F*^ with an aim to reducing the clinical burden of thrombosis in MPN patients post diagnosis. Both interferon and the *JAK2* inhibitor ruxolitinib have been shown to reduce *JAK2*^*V617F*^ VAF in the RESPONSE and PROUD-PV trials [18, 19]. Future trials addressing clonal expansion of *JAK2*^*V617F*^ may provide further opportunities to improve outcomes, prognostication and monitoring in patients with MPNs.

We observed a higher rate of thrombotic events in patients with PV when compared with ET and PMF (Table 3). This result is consistent with previous studies and is likely a result of higher *JAK2*^*V617F*^ VAF, WCCs and erythrocytes in PV, all of which have been correlated with increased thrombotic risk [20, 21]. Interestingly, raised platelet counts have not been independently associated with risk of thrombosis; on the contrary, platelets counts above 1500 are associated with increased risk of bleeding as a result of the clearance of von Willebrand factor (VWF) multimers [22, 23].

We recognise limitations in this retrospective study, such as the data regarding thrombotic events. This introduces a degree of uncertainty as thrombotic events could not be independently verified and may be impacted by the lack of clinical record or patients’ inability to precisely recollect their past medical history. Previous studies have shown a correlation between *JAK2*^*V617F*^ VAF and venous but not arterial thrombosis. We observed no associations between venous or arterial thrombosis and *JAK2*^*V617F*^ VAF when taken independently (data not shown). Our low patient number with venous thrombosis may limit our ability to identify this association. Moreover, the cohort only included a small number of patients diagnosed with PMF. As such, caution must be taken when drawing conclusions from this study data regarding *JAK2*^*V617F*^ VAF and its prognostic impact in PMF.

## Conclusion

One hundred and fifty-nine patients with MPNs were included in this study, and their demographic and clinical characteristics as well as *JAK2*^V617F^ VAF were determined. We have shown that higher *JAK2*^V617F^ VAF is associated with thrombotic events in our MPN cohort and that this is primarily due to higher *JAK2*^V617F^ VAF in MPN patients that experienced thrombotic events post diagnosis. These results suggest that *JAK2*^V617F^ VAF may provide a prognostic indicator for risk of developing thrombotic events post diagnosis.

## Data Availability

The datasets used or analysed during the current study are available from the corresponding author upon reasonable request.

## Declarations

### Provence and Peer review

Not commissioned; externally peer reviewed.
